# The q-Dixon sequence for MRI predicts osteoporotic vertebral compression fractures

**DOI:** 10.3389/fendo.2025.1591044

**Published:** 2025-08-25

**Authors:** Jing Zhang, Qiyuan Li, Yao Wang, Li Sun, Qingyuan Zhang, Chuanping Gao

**Affiliations:** Department of Radiology, The Affiliated Hospital of Qingdao University, Qingdao, Shandong, China

**Keywords:** magnetic resonance imaging, osteoporosis, vertebral compression fracture, fat fraction, bone mineral density

## Abstract

**Objectives:**

To evaluate whether q-Dixon sequence-based fat fraction (FF) values of the lumbar spine can predict osteoporotic vertebral compression fracture (OVCF) risk in older adult(s) osteoporosis patients.

**Materials & methods:**

Thirty OVCF patients and 15 osteoporosis patients were enrolled. Areas of interest (ROIs) were manually drawn using the post-processing workstation, and FF values of the patient’s L1–L4 vertebrae (except the fractured vertebrae) were measured. The Pearson correlation test was used to analyze the correlation between the average lumbar spine FF value and bone mineral density (BMD). The receiver operating characteristic curve (ROC) and area under the curve (AUC) were used to evaluate the prediction efficiency of the FF values and to determine the best cut-off value for prediction.

**Results:**

The average lumbar spine FF value and the FF values of the L1–L4 vertebrae in the fracture group were significantly higher than those in the non-fracture group, and there was no significant difference in BMD between the two groups. ROC analysis showed that the AUC of the average lumbar spine FF value was 0.822, with sensitivity = 73.3%, specificity = 86.7%, and cut-off value = 57.27%. Among the L1–L4 vertebrae, the FF value of L2 vertebrae had the highest AUC of 0.870, and the cutoff value was 56.62%.

**Conclusion:**

The FF values of the lumbar spine measured by the q-Dixon sequence can help predict OVCF risk and provide complementary information to BMD measurements. The FF value of L2 vertebrae has the best prediction efficiency. However, these findings should be interpreted with caution given the relatively small sample size (n=45) and manual ROI segmentation method used in this study

## Introduction

Osteoporotic vertebral compression fracture (OVCF) is the most common osteoporotic fracture in the clinic and can occur in response to slight external force under conditions of decreased bone mass and bone strength and increased bone fragility (1). According to the International Osteoporosis Foundation, approximately 50% of women and 25% of men worldwide are expected to experience at least one osteoporotic fracture during the latter half of their lives (2). As the population ages, OVCFs are increasingly affecting the older adult(s), decreasing patients’ ability to perform many routine activities of daily living and even causing patient death. Therefore, it is important to identify individuals at high risk of OVCF among osteoporosis patients, to predict fracture risk, and to initiate early intervention.

Dual energy X-ray absorptiometry (DXA) is widely used in the diagnosis and evaluation of osteoporosis (3). However, BMD is representative of only 50%–70% of bone strength, and it exposes patients to radiation (4). Therefore, alternative radiation-free imaging techniques and biomarkers need to be investigated. Despite these limitations, DXA remains the clinical gold standard due to its low cost, widespread availability, and rapid acquisition time. Studies have shown that an increase in fat cells in the bone marrow is accompanied by a decrease in osteoblasts and that fat tissue is used to “fill” the extra bone marrow space when bone mass is reduced (5). Thus, the onset of osteoporosis is associated with an increase in bone marrow fat mass.

Chemical shift-encoding water-fat imaging (Dixon) is an effective method for rapid and non-invasive assessment of the bone marrow fat content and was first proposed by Dixon in 1984 (6). Initially, the two-point Dixon sequence was proposed, wherein the hydrogen proton signals in water and fat are collected when they are in the in-phase (IP) and opposed-phase (OP), respectively, by setting different echo times and calculating the water–fat separation images. The main limitation of this method is its sensitivity to magnetic field inhomogeneity. To address this problem, Glover and Schneider proposed the three-point Dixon sequence (7). Along with the advances in MR technology, various fat content quantification sequences based on the three-point Dixon method have been developed, including Ideal IQ (GE Healthcare), mDixon (Philips Healthcare), and q-Dixon (Siemens Healthcare). In recent years, the modified Dixon sequence has been increasingly applied in the quantitative analysis of fat in vertebrae.

We hypothesize that: (1) FF values will be significantly higher in patients with OVCF compared to those without fractures, and (2) FF measurements will provide fracture risk prediction independent of BMD. In this study, the q-Dixon sequence was used to measure the fat fraction (FF) value of the lumbar spine in osteoporosis patients. We hypothesized that FF values may predict OVCF to a certain extent.

## Materials and methods

### Patients

This prospective study was conducted in accordance with the Declaration of Helsinki (as revised in 2013). The study was approved by the institutional Review Board of the Affiliated Hospital of Qingdao University, and informed consent was obtained from all patients.

A total of 30 older adult(s) patients presenting with OVCF (fracture group) and 15 older adult(s) patients diagnosed with osteoporosis during the same period (non-fracture group) were enrolled from July 2022 to December 2023. The sample size of 45 patients (30 fracture, 15 control) was determined based on clinical availability rather than formal power calculation, which may limit the generalizability of our findings. Patients were consecutively enrolled without randomization to reflect real-world clinical practice. All participants provided written informed consent within 24 hours before undergoing MRI and BMD assessments. All patients were examined within a week of diagnosis, and MRI and BMD examinations were performed on the same day.

The inclusion criteria for the fracture group were as follows: (1) no history of fragility fractures; (2) osteoporosis confirmed by BMD measurement; and (3) fresh vertebral fracture identified on MRI. Fresh fracture was defined as acute vertebral collapse with MRI evidence of bone marrow edema (T1 hypointensity, T2/STIR hyperintensity) and symptom onset within ≤4 weeks. The exclusion criteria were as follows: (1) poor image quality; (2) number of fractured lumbar vertebrae ≥ 2; (3) compression fracture caused by high-energy trauma; (4) anti-osteoporosis therapy prior to MRI and BMD measurement; (5) presenting with other diseases that have the potential to impact bone quality; (6) and taking drugs that could affect bone metabolism.

The inclusion criteria for the non-fracture group were as follows: (1) osteoporosis was confirmed by BMD measurement; and (2) imaging studies did not show any signs of fragility fractures. The exclusion criteria were as follows: (1) poor image quality; (2) anti-osteoporosis therapy prior to MRI and BMD measurement; (3) presenting with other diseases that have the potential to impact bone quality; and (4) taking drugs that could affect bone metabolism.

**Figure 1:**
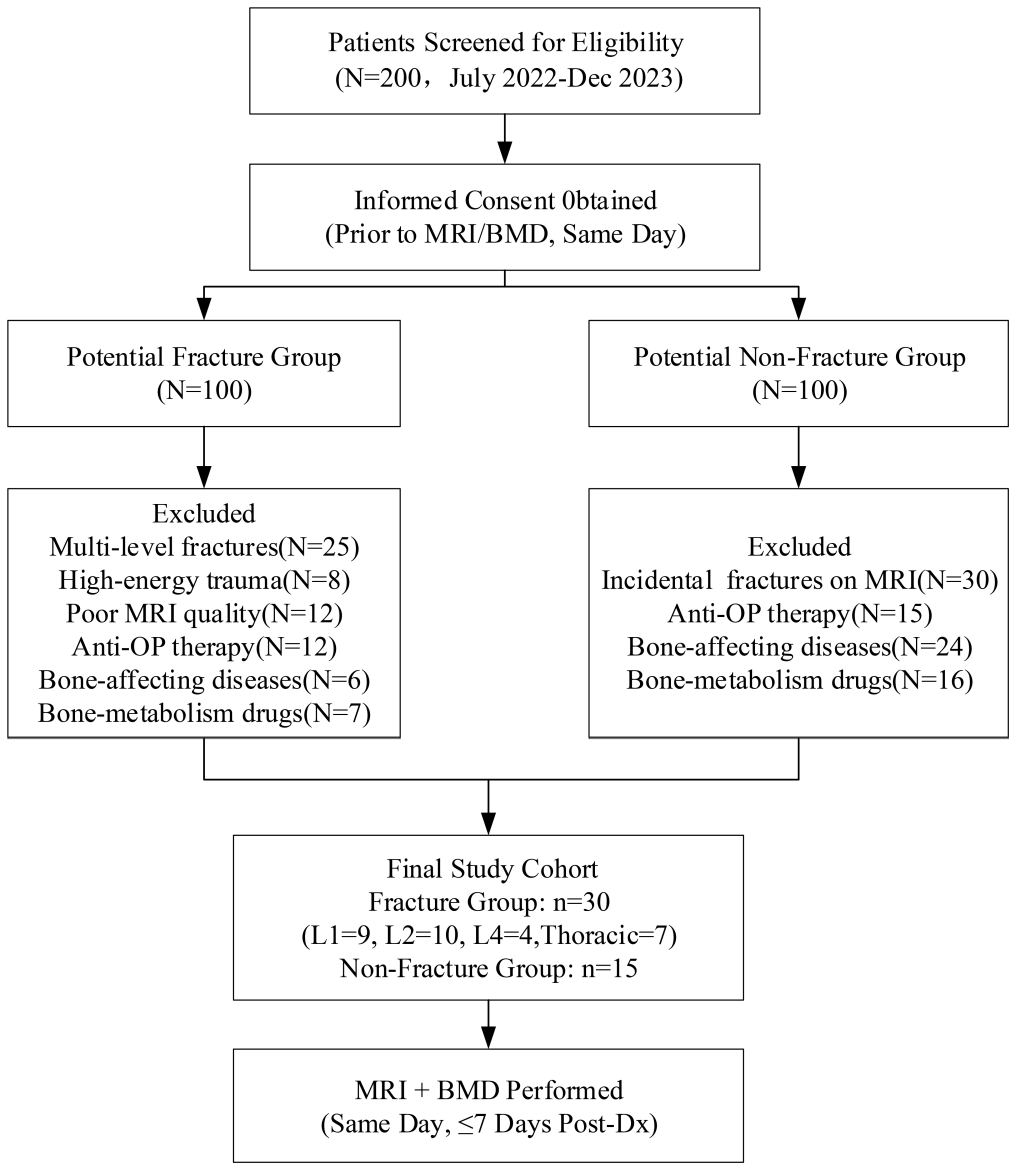
Flowchart of patient selection and study enrollment.

**Figure 1** illustrates the patient enrollment process. From an initial screening cohort of 200 eligible candidates, 45 participants met the final inclusion criteria, comprising 30 patients with acute osteoporotic vertebral compression fractures (OVCF) and 15 age-matched osteoporosis controls without vertebral fractures.

### MRI examination

MRI was performed using a Prisma 3.0T MRI system (Siemens Healthcare GmbH, Erlangen, Germany) with an 18-channel body phased-array coil. The examination site was the lumbar spine. Anatomical and morphological evaluation of the vertebrae was obtained using conventional T1WI (sagittal, repetition time [TR]/echo time [TE] = 400/20 ms, field of view [FOV] = 300 × 300 mm^2^, matrix size = 240 × 320, slice thickness = 4.0 mm, gap = 1.0 mm, and acquisition time = 124 s) and T2WI (sagittal, TR/TE = 2700/119 ms, FOV = 300 × 300 mm^2^, matrix size = 288 × 384, slice thickness = 4.0 mm, gap =1.0 mm, and acquisition time = 118 s). The fat mass of the vertebrae was obtained using the q-Dixon sequence (sagittal, TR/TE = 9.40/1.29 ms, FOV = 380 × 380 mm^2^, matrix size = 96 × 96, slice thickness = 3.0 mm, gap = 1.0 mm, and acquisition time =108 s).

### Imaging analysis

**Figure 2:**
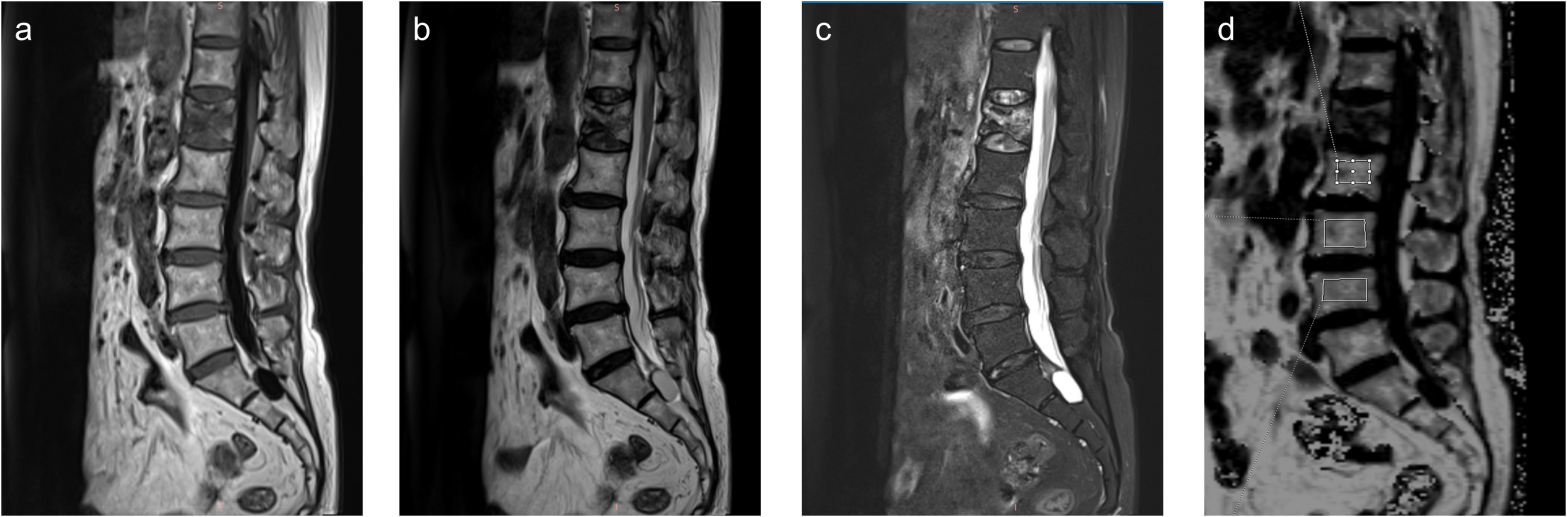
Sagittal T1-weighted **(a)**, T2-weighted **(b)**, Fat-sat T2-weighted **(c)** images and fat fraction (FF) map **(d)** of a 73-year-old female patient with an osteoporotic vertebral compression fracture in L1 vertebrae.

Post-processing was performed on a Syngo.via workstation. The sagittal FF map was automatically generated and transmitted to the workstation after the q-Dixon sequence. Rectangular regions of interest (ROIs) were manually drawn on the FF map by two radiologists experienced in musculoskeletal radiology (**Figure 2**). Manual ROI placement was performed by two experienced radiologists to accommodate anatomical variations, though this approach may introduce more subjectivity compared to automated segmentation methods. During the segmentation, cortical bone and the vertebral endplate were avoided as much as possible, and vertebrae with fractures or degenerative changes (e.g., Modic changes) were excluded. At least three vertebrae were measured for each patient. Each vertebra was measured three times and averaged. The average of the two radiologists’ measurements was taken as the final result. After measurement, the FF values of the L1–L4 vertebrae of each patient were recorded, and the average FF values of the lumbar spine were calculated.

### BMD measurement

Bone mineral density (BMD) was quantified using dual-energy X-ray absorptiometry (DXA; Osteosys Primus, Seoul, Korea) following standardized protocols. Scans were performed by certified technologists at two anatomical sites: (1) lumbar spine (L1-L4 vertebrae) and (2) right proximal femur. The system was calibrated daily using manufacturer-provided phantoms, with a demonstrated coefficient of variation <1% for lumbar spine measurements.For each participant:(1)Areal BMD (g/cm²) was automatically calculated for all measurable vertebrae.(2)Fractured vertebrae were systematically excluded from lumbar spine analysis.(3)T-scores were derived using the results of BMD studies in normal individuals.(4)Scan quality was verified by a musculoskeletal radiologist blinded to clinical data.

### Statistical analysis

SPSS 26.0 and MedCalc 22.016 software were used for statistical calculations with a two-sided level of significance of 0.05. The Shapiro–Wilk test was used to assess the normality of the data. Student’s t-test or chi-square test was used to analyze the data with a normal distribution. The Mann–Whitney U test was used to analyze non-normally distributed data. The intraclass correlation coefficient (ICC) was used to evaluate the consistency of the FF values measured by the two radiologists. An ICC > 0.80 was considered good consistency. The Pearson correlation coefficient was used to evaluate the correlation between the FF value and BMD. The receiver operating characteristic curve (ROC) was plotted, and the area under the curve (AUC), sensitivity, and specificity were calculated to assess the predictive value of the FF value for OVCF. The cut-off value of the FF value was determined using the Youden index, which was calculated as: sensitivity + specificity − 1.

## Results

**Table 1** shows the general characteristics of patients in the fracture and non-fracture groups. The age range of the fracture group was 53–87 years (66.67 ± 7.30), and the age range of the non-fracture group was 52–87 years (68.33 ± 8.96). No significant differences in age, sex, height, weight, and BMI were found between the two groups (P > 0.05). In the fracture group, there were 9 fractures in the L1 vertebrae, 10 in the L2 vertebrae, 4 in the L4 vertebrae, and 7 in the thoracic vertebrae, including 1 in the T8 vertebrae, 2 in the T11 vertebrae, and 3 in the T12 vertebrae.

**Table 1:** Patients characteristics.

Clinical characteristics	Fracture group (n=30)	Non-fracture group (n=15)	P
Age (Mean ± SD), year	66.67 ± 7.30	68.33 ± 8.96	0.507
Sex (Male/Female)	21/8	10/5	0.692
Height (Mean ± SD), cm	160.33 ± 7.57	159.80 ± 4.75	0.850
Weight (Mean ± SD), Kg	62.80 ± 9.73	61.33 ± 7.79	0.614
BMI (Mean ± SD)	24.23 ± 3.24	23.94 ± 2.87	0.768

SD, Standard Deviation; BMI, Body mass index.

**Figure 3:**
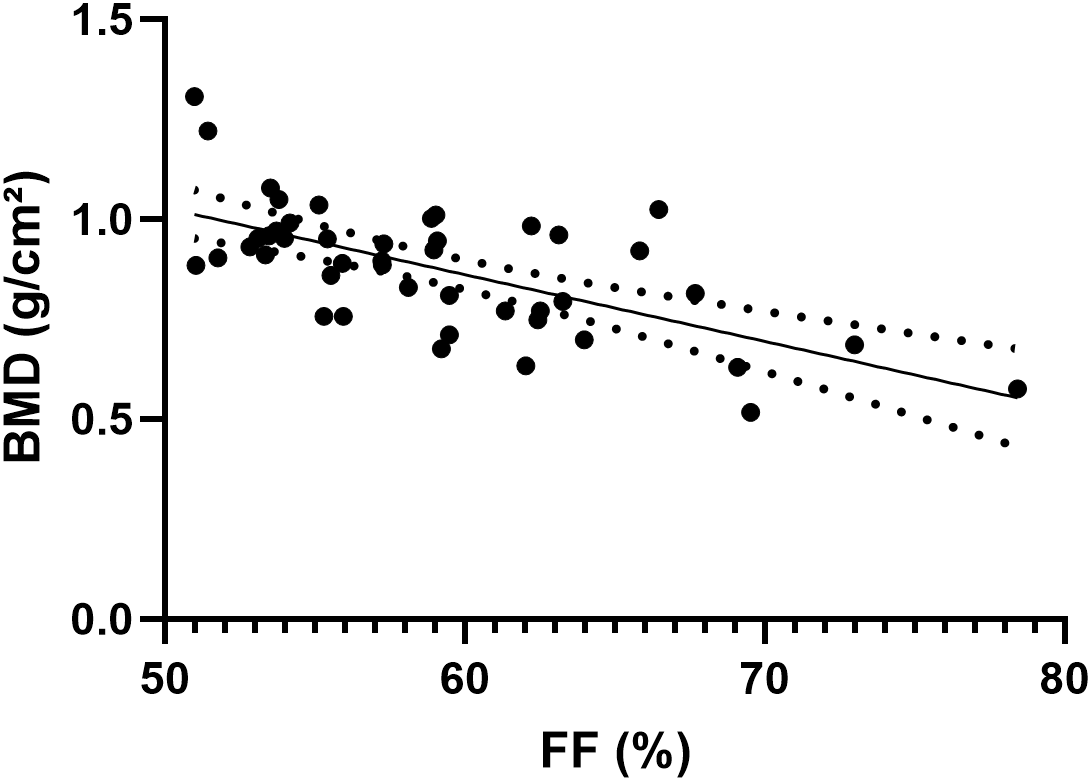
Scatter plot of the correlation between the average FF value of the lumbar spine and BMD.

The consistency in the FF values of the L1–L4 vertebrae measured by the two radiologists was excellent, with ICC values of 0.996 (95% confidence interval, 0.995–0.997). **Figure 3** shows the results of the Pearson correlation analysis between the average FF value of the lumbar spine and BMD in the two groups. A significant negative correlation was observed between the average lumbar spine FF value and BMD (r = −0.6489, P < 0.001).

**Table 2**. Comparison of vertebral fat fraction (FF) values between fracture and non-fracture groups and **Figure 4** show that the average lumbar spine FF value and the FF values of the L1–L4 vertebrae in the fracture group were significantly higher than those in the non-fracture group. The FF values in the two groups increased from the L1 to L4 vertebrae, and the difference was statistically significant (P < 0.05). There was no significant difference in BMD between the two groups (P > 0.05). The ROC curves of the average FF value of the lumbar spine and the FF values of the L1–L4 vertebrae are shown in **Figure 5**; **Table 3** shows the AUC, sensitivity, specificity, cut-off value, and other parameters of the lumbar spine FF value. The FF value of the L2 vertebrae had the highest AUC of 0.870, with a sensitivity and specificity of 80%, and its cut-off value was 56.62%. The superior predictive performance at L2 (AUC=0.870) may reflect its transitional biomechanical position in the spine, experiencing both compressive and shear forces that make it particularly sensitive to marrow composition changes. The FF value of the L3 vertebrae had the highest specificity of 100%, but the AUC was only 0.752, and the cut-off value was 61.27%. The AUC of the average FF value of the lumbar spine was 0.822, with a sensitivity of 73.3%, specificity of 86.7%, and cut-off value of 57.27%.

**Table 2:** Differences in BMD and FF values between fracture group and non-fracture group .

Clinical characteristics	Fracture group (n=30)	Non-fracture group (n=15)	P
BMD	0.852 ± 0.15	0.929 ± 0.16	>0.05
FF (%)	61.08 ± 6.27	54.84 ± 3.09	0.001
L1-FF (%)	58.52 ± 7.86	53.11 ± 2.92	0.017
L2-FF (%)	61.63 ± 7.21	54.35 ± 3.38	0.001
L3-FF (%)	61.76 ± 6.59	56.21 ± 2.97	0.004
L4-FF (%)	62.38 ± 6.56	55.71 ± 3.91	0.001

BMD, Bone mineral density; FF, Fat fraction.

**Figure 4:**
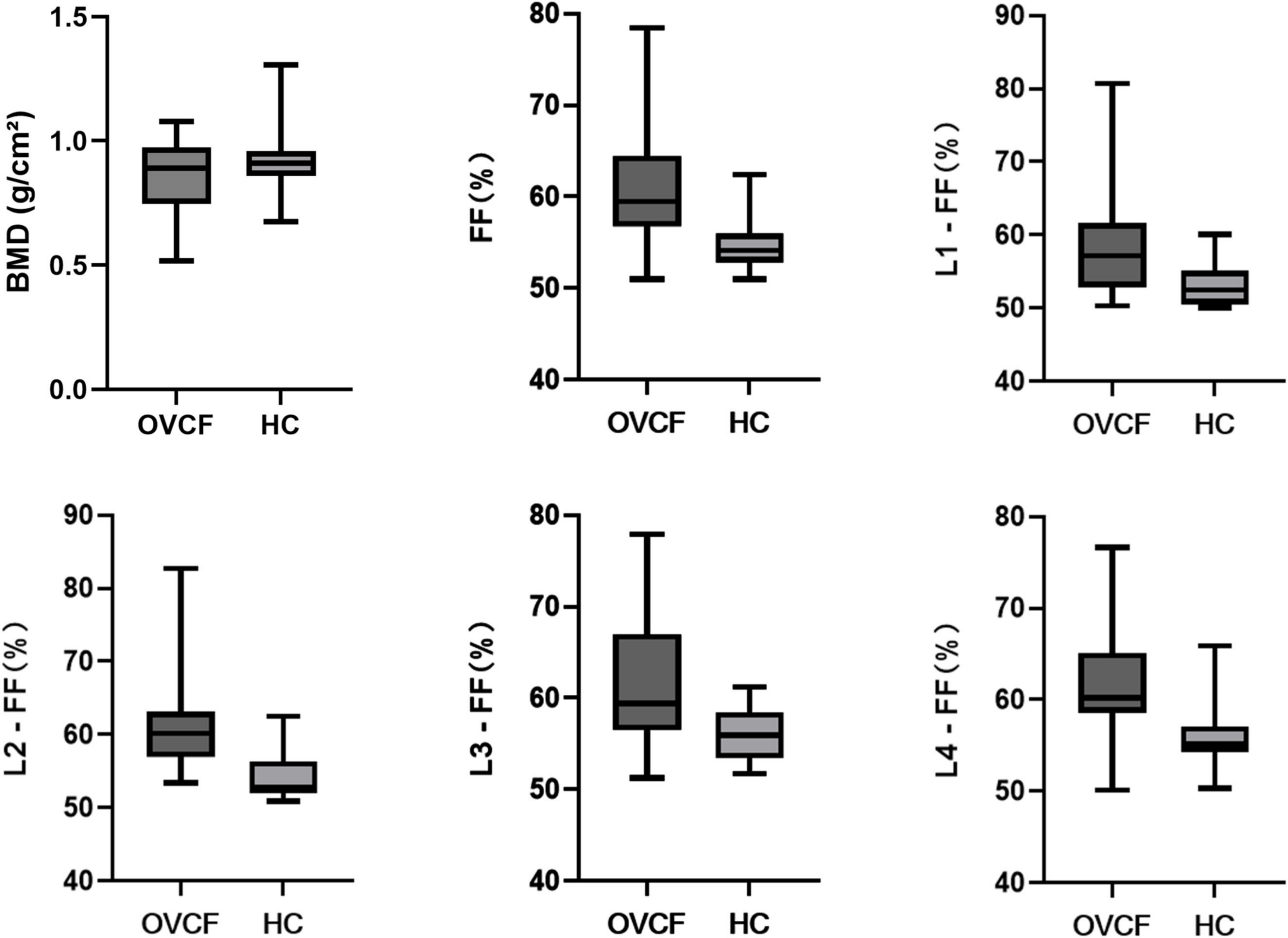
Analysis of the differences between the fracture group and the non-fracture group. OVCF: fracture group; HC: non-fracture group.

**Figure 5:**
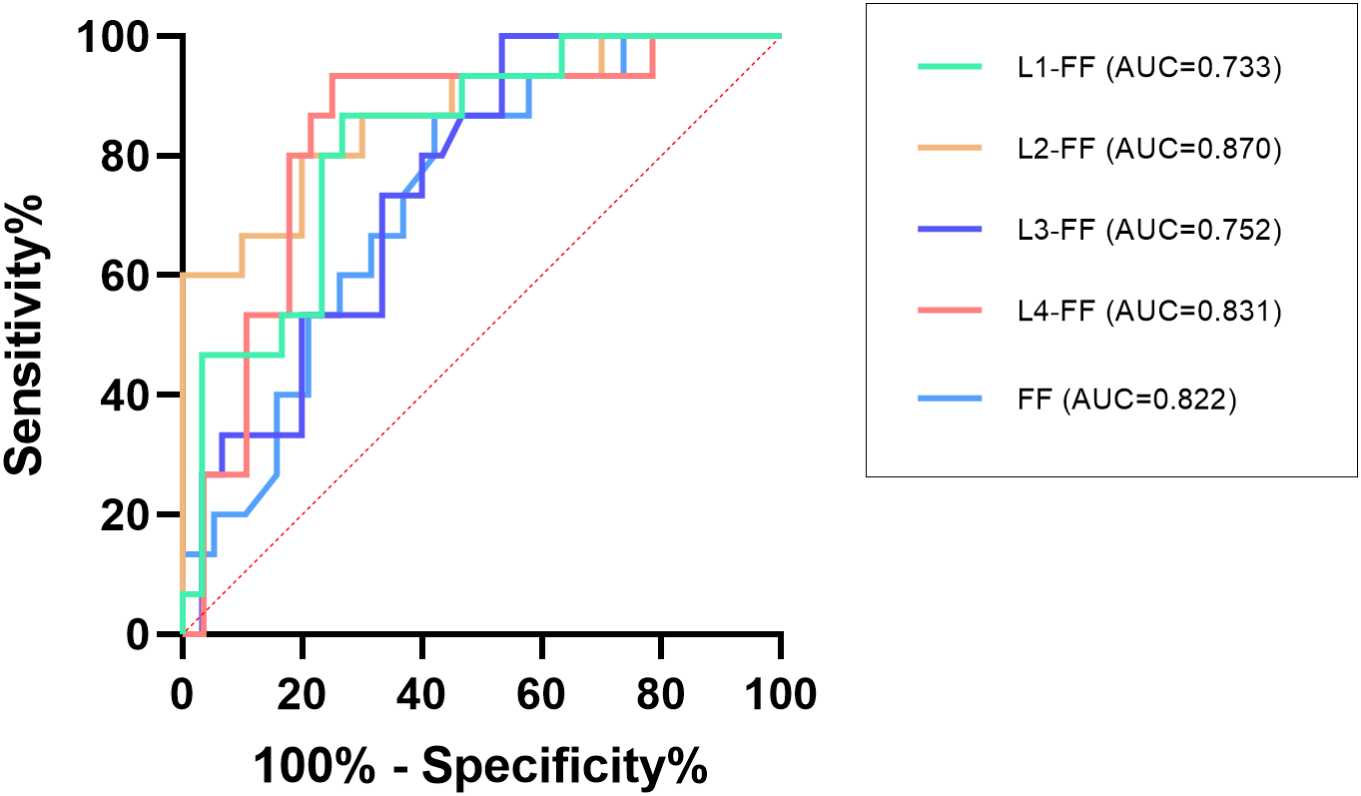
ROC analysis of the average FF value of the lumbar spine and the FF values of the L1–L4 vertebrae.

**Table 3:** Performance of the parameters for predicting OVCF .

Clinical characteristics	AUC	Cut-off value (%)	95% CI	SEN (%)	SPE (%)	P	Youden-index
FF (%)	0.822	57.27	(0.697-0.947)	73.30	86.70	0.001	0.600
L1-FF (%)	0.733	55.74	(0.565-0.902)	57.90	86.70	0.021	0.446
L2-FF (%)	0.870	56.62	(0.749-0.991)	80.00	80.00	0.001	0.600
L3-FF (%)	0.752	61.27	(0.612-0.893)	47.60	100.00	0.006	0.467
L4-FF (%)	0.831	59.28	(0.699-0.963)	75.00	93.30	0.001	0.683

AUC, Area under the curve; CI, Confidence interval; SEN, Sensitivity; SPE, Specificity.

## Discussion

In this study, we evaluated the ability of the q-Dixon sequence to measure the lumbar fat mass in osteoporosis patients and explored its feasibility for predicting OVCF. The q-Dixon sequence is based on chemical shifts, which exploit the differential separation of proton resonance frequencies in water and fat to achieve high-resolution and high-contrast images of tissue structure (6). This technology can collect six echoes at one time and can accurately identify the water and fat signals in human tissue using the 7-peak fat model and T2*correction, with high spatial resolution and convenient operation (8). The findings indicate that the q-Dixon sequence is effective in predicting OVCF risk, with the highest predictive efficacy in the L2 vertebral FF measurement. While we interpret elevated FF values as a predisposing factor for fracture, we acknowledge that bone marrow fat is dynamic. Although FF changes typically develop over weeks to months, post-fracture immobility or inflammatory responses could theoretically influence FF measurements even within the first week after fracture.This aligns with broader research that emphasizes the importance of accurate, early OVCF detection to prevent adverse outcomes and manage the healthcare burden.

A meta-analysis showed that BMD reduction could explain approximately 70% of the risk of osteoporotic fractures (9). For postmenopausal osteoporosis patients, a 1-standard deviation decrease in BMD was associated with a 1.5–2.0-fold increase in fracture risk (10). However, BMD has some limitations in fracture risk assessment. Most patients with osteoporotic fractures have a BMD that is not within the range of diagnosis of osteoporosis (11–13). Therefore, factors related to bone mass should be considered in the assessment of patients with osteoporotic fractures. Bone marrow fat is considered an important biological marker for bone quality assessment (14, 15). As bone grows and ages, the number of adipocytes in bone marrow gradually increases, which is closely related to bone metabolism. The only difference is that bone mass continues to increase during growth and sexual maturity, whereas as the body ages, sex hormone activity decreases, resulting in bone mass loss (16). Adipocytes and osteoblasts in the bone marrow originate from the same precursor, namely, marrow mesenchymal stem cells (MSCs). MSCs have multilineage differentiation potential. Competition exists between adipocytes and osteoblasts, which are in a dynamic equilibrium state. A decrease in osteoblasts tends to be accompanied by an increase in adipocytes (17, 18). Moreover, adipocytes secrete saturated fatty acids. Saturated fatty acids have been suggested to possibly have a lipotoxic effect on osteoblasts, thereby affecting their normal function (19). Previous studies have shown that changes in the bone marrow fat precede changes in bone mass during the development of osteoporosis (20, 21).

Our findings demonstrate that FF provides complementary information to BMD, with the inverse correlation (r=-0.6489) suggesting FF captures distinct aspects of bone quality. This supports emerging paradigms of osteoporosis as both a quantitative (BMD) and qualitative (marrow composition) disorder of bone (17, 21, 22). The average lumbar spine FF value gradually increased with a decrease in BMD, reflecting the competition between adipogenesis and osteogenesis in bone marrow, in agreement with the conclusions of previous studies (22, 23). The increase in the fat content may indirectly reflect a decrease in bone mass and change in bone strength. The reasons for these results may include the following (24–27): (1) the balance between adipocyte and osteoblast differentiation is disrupted, which affects bone strength; (2) saturated fatty acids secreted by adipocytes have lipotoxic effects on osteoblasts; (3) fat accumulation can replace functional hematopoietic cells and osteoblasts because of the restricted space within the bone marrow; and (4) with an increase in age, the levels of inhibitory factors such as estrogen and transforming growth factor decrease, which is not conducive to osteoblast differentiation.

The average FF value of the lumbar spine and the FF values of the L1–L4 vertebrae in patients with fracture were higher than those in patients without fractures, and there was no significant difference in BMD between the two groups. This finding is similar to that of Gassert et al. (28), who evaluated the ability of the proton density fat fraction (PDFF) of vertebral bone marrow to differentiate between osteoporotic/osteopenic patients with and without vertebral compression fractures. The results showed that patients with fractures had a significantly higher PDFF than those without fractures after adjusting for clinical factors (P < 0.001), although BMD showed no significant differences among the subgroups. This effect is not just simply caused by the strong correlation between the FF values and BMD. The FF values should be considered as an important component in the assessment of bone fragility to assess fracture risk in osteoporosis patients. Furthermore, consistent with previous studies (29, 30), the vertebral FF values increased from L1 to L4, reflecting the transformation of the bone marrow from red to yellow with age, and from peripheral to axial, but the pathophysiological relevance of this finding is unclear.

The ROC curve showed that the FF value of the L2 vertebrae had the best performance in predicting OVCF (AUC = 0.870, sensitivity = 80%, and specificity = 80%), the FF value of the L3 vertebrae had the highest specificity of 100% (AUC = 0.752). This variation in diagnostic performance across vertebral levels may reflect anatomical differences in biomechanical loading patterns, regional variations in marrow composition, or statistical variability due to sample size limitations. The AUC of the average FF value of the lumbar spine was 0.822 (sensitivity = 73.3%, specificity = 86.7%), and the cutoff value was 57.27%. The combination of multiple vertebrae was likely to result in reduced predictive sensitivity. Overall, the average FF value of the lumbar spine showed stable ability in predicting OVCF. Therefore, FF values can be considered as a complement to BMD assessment of bone strength in the risk assessment of vertebral fractures in osteoporosis patients. Yun et al. conducted a similar study and reported a lower cut-off value of 42% (31); Gassert et al. calculated a cut-off value of 44.9% (28). Potential reasons for why the cutoff value in this study is higher than those reported in similar studies may include the following. (1) Individual differences exist in the samples, such as the inclusion criteria and age range of the samples. Unlike most previous studies, which were retrospective and included a wide age range of patients, this study was a prospective study of older adult(s) patients with osteoporosis who had high levels of bone marrow fat. (2) Group differences such as sex, region, and race need to be confirmed by expanding the sample size and performing further epidemiological investigation. (3) Differences in technical metrics, such as the MR scanners used in different studies and the technical principles of the MR scanners, may lead to differences in the results.

Accurate prediction of osteoporotic fracture risk can be of significant clinical benefit in the assessment and management of osteoporosis. Beyond its predictive value, FF measurement offers unique advantages as it reflects aspects of bone quality not captured by BMD. Importantly, FF is modifiable - studies demonstrate that interventions including bisphosphonates, teriparatide, exercise, and hormonal therapy can reduce marrow fat content (17, 21, 22). This suggests FF may serve as both a predictive biomarker and a treatment response indicator. The FF value is readily available clinically and has good predictive efficacy, which can help clinicians distinguish between high- and low-risk groups of OVCF to formulate corresponding treatment plans. When a patient’s lumbar spine FF value is close to or above the cut-off value, intervention at multiple levels such as lifestyle, diet, exercise, and medication is necessary, with particular attention focused on preventing falls or injuries to avoid or delay the occurrence of OVCF.

Several limitations must be acknowledged: (1) The cross-sectional design precludes causal inferences about FF and fracture risk; (2) Generalizability may be limited by the homogeneous sample (older adult(s) Asian population) - validation in diverse ethnic groups and younger patients is needed (32); (3) Manual ROI segmentation, while clinically practical, introduces measurement variability versus automated methods; (4) The exact temporal relationship between fracture onset and FF measurement remains unclear;(5)The sample size is relatively small, and larger cohort studies are needed to validate and revise the results of this study; (6) while MRI was performed within one week of diagnosis, the exact interval between fracture onset and imaging was not recorded. This temporal relationship is important to confirm that FF changes preceded rather than resulted from the fracture. (7) Our study used DXA, which is routinely used in clinical practice to assess BMD, but a study has shown that standardized QCT was more accurate (26). Future research should focus on optimizing the measurement techniques to improve the accuracy of the data; (8)The ROIs were manually determined, which means that the shapes may not be in perfect agreement and may lead to errors. Therefore, several measurements were performed in this study, and the average was used as the final result.(9) While we controlled for age statistically, the modest sample size precluded meaningful age-stratified analyses. Future larger studies should examine age-specific FF thresholds.

## Conclusion

In summary, our study shows that FF values of lumbar spine measured using the q-Dixon sequence are helpful in predicting the risk of vertebral compression fractures in osteoporosis patients. While promising, the q-Dixon sequence may become a practical tool for non-invasive assessment of fracture risk, though its clinical implementation requires validation in larger, prospective studies with standardized protocols across diverse populations.

## Data Availability

The original contributions presented in the study are included in the article/supplementary material. Further inquiries can be directed to the corresponding author.
